# Synergistic Enhancement of Thermoelectric Performances by Cl-Doping and Pb-Excess in (Pb,Sn)Se Topological Crystal Insulator

**DOI:** 10.3390/ma14081920

**Published:** 2021-04-12

**Authors:** Jin Hee Kim, Gareoung Kim, Seokyeong Byeon, Hyungyu Jin, Jong-Soo Rhyee

**Affiliations:** 1 Department of Applied Physics, Integrated Education Institute for Frontier Science and Technology (BK21 Four) and Institute of Natural Sciences, Kyung Hee University, Yongin 17104, Korea; 2 Energy Materials Laboratory, Toyota Technological Institute, Nagoya 468-8511, Japan; noah04@naver.com; 3 Department of Mechanical Engineering, Pohang University of Science and Technology (POSTECH), Pohang 37673, Korea; sybyeon@postech.ac.kr (S.B.); hgjin@postech.ac.kr (H.J.)

**Keywords:** (Pb,Sn)Se, crystalline mirror symmetry breaking, Cl-doping, Pb-excess, thermoelectric

## Abstract

We investigated the thermoelectric properties of the Pb_0.75_Sn_0.25_Se and Pb_0.79_Sn_0.25_Se_1−x_Cl_x_ (x = 0.0, 0.2, 0.3, 0.5, 1.0, 2.0 mol.%) compounds, synthesized by hot-press sintering. The electrical transport properties showed that low concentration doping of Cl (below 0.3 mol.%) in the Pb-excess (Pb,Sn)Se samples increased the carrier concentration and the Hall mobility by the increase of carriers’ mean free path. The effective mass of the carrier was also enhanced from the measurements of the Seebeck coefficient. The enhanced effective masses of the carrier by the Cl-doping can be understood by the enhanced electron-phonon interaction, caused by the crystalline mirror symmetry breaking. The significantly decreased lattice thermal conductivities showed that the crystalline mirror symmetry breaking decreased the lattice thermal conductivity of the Pb-excess (Pb,Sn)Se. By the Cl-doping and the Pb-excess’s synergistic effect, which can suppress the bipolar effect, the *zT* values of x = 0.2 and 0.3 mol.% reached 0.8 at 773 K. Therefore, we suggest that Pb-excess and the crystalline mirror symmetry breaking by Cl-doping are effective for high thermoelectric performance in the (Pb,Sn)Se.

## 1. Introduction

Thermoelectric devices based on the Seebeck, Peltier, and Thomson effects can be used for thermoelectric power generation or solid-state cooling, including flexible or wearable thermoelectric devices [1,2]. The thermoelectric performance of the device is mainly determined by the dimensionless thermoelectric figure of merit (*zT*), which is defined by *zT = S^2^σT/κ*, where *S*, *σ*, *T*, and *κ* are the Seebeck coefficient, electrical conductivity, absolute temperature, and thermal conductivity, respectively. The high-performance thermoelectric materials (Bi_2_Te_3_, Bi_2_Se_3_, Sb_2_Te_3_, SnTe, etc.) have been revealed to have topologically protected states such as the topological insulator (TI) or the topological crystalline insulator (TCI) states, etc. [3].

The PbTe is the thermoelectric material with high *zT* values in both the *n-*type (1.8 at 773 K [4]) and *p-*type (2.5 at 923 K [5]). Even though the PbTe has good thermoelectric properties, the low abundance of tellurium in the earth’s crust is one of the disadvantages of practical thermoelectric applications [6]. Because of the abundance of Se compared with the Te and the similar crystal structure of PbSe compared with PbTe, the PbSe is a promising candidate to replace the PbTe. Furthermore, recent investigations show that the thermoelectric performance of the *n-*type PbSe are comparable with those of the PbTe [6].

The PbSe shows a topological phase transition from a trivial insulator to a TCI by Sn substitution at low temperature [7]. The TCI is one of the topological states caused by crystalline mirror symmetry and the mirror Chern number. The theoretical calculation shows that the breaking of the TCI state by lowering crystal symmetry can increase the thermoelectric power factor [8]. The crystal symmetry of the TCI state can be broken by various perturbations such as the application of electric fields [9], mechanical strain [10], chemical doping [11,12,13], etc. The chemical doping enlarges the bandgap energy by the mirror symmetry breaking with the control of the carrier concentration. For example, Na-doping in the TCI Pb_0.6_Sn_0.4_Te shows the enhancement of thermoelectric performance by the crystalline mirror symmetry breaking with the increased bandgap from ~0.1 to~0.22 eV, as well as the optimization of the carrier concentration [12]. Moreover, Cl-doping in TCI Pb_0.7_Sn_0.3_Se enhances the bulk bandgap, controls the carrier concentration, and decreases the lattice thermal conductivity [13].

Additionally, the Pb excess in the PbSe can increase the thermoelectric performance of the *n-*type PbSe by decreasing the intrinsic defects, which can cause the generation of hole carriers [14]. The intrinsic defects study using a first-principles density-functional theory shows that the rock salt Pb chalcogenides (PbS, PbSe, and PbTe) have intrinsic Pb vacancy and Schottky defects, which play as acceptors and donor-acceptor pairs, respectively [15]. Experimentally, the hole carrier reduction of the PbSe by the Pb-excess is proved by showing that the Seebeck coefficients of the PbSe are changed from positive values to negative values by the Pb-excess [14].

This study investigates the thermoelectric properties of the Cl-doped Pb_0.79_Sn_0.25_Se compounds and the non-Pb-excess sample (Pb_0.75_Sn_0.25_Se). The Cl-doping can increase the Seebeck coefficient by the enhanced effective mass of the carrier with the increase of the electronic bandgap [13], and the non-stoichiometric Pb addition can reduce the intrinsic defects of the PbSe [14]. However, there are not many studies about whether the Cl-doping can influence the Pb-excess (Pb,Sn)Se compound or not. Therefore, herein we present the Cl-doping and Pb-excess’s synergistic effect in (Pb,Sn)Se toward high thermoelectric performance.

## 2. Materials and Methods

The sintered bulk samples of the Pb_0.79_Sn_0.25_Se_1−x_Cl_x_ (x = 0.0, 0.2, 0.3, 0.5, 1.0, 2.0 mol.%) and Pb_0.75_Sn_0.25_Se were prepared by the conventional melting and hot-press sintering method. The high-purity elements of the Pb (99.999%, RND Korea, Gwangmyeong, Korea), Sn (99.999%, RND Korea, Gwangmyeong, Korea), Se (99.999%, RND Korea, Gwangmyeong, Korea), and PbCl_2_ (99.99%, RND Korea, Gwangmyeong, Korea) were loaded into evacuated quartz tubes (HANJIN QUARTZ, Seoul, Korea). The quartz tubes were sealed under the high vacuum and heated up to 1373 K for 1 day (Vacuum sealing machine, Y&I Tech, Paju, Korea). The sealed quartz tubes were quenched and annealed at 823 K for 2 days. The obtained ingot samples were pulverized using an agate mortar (LKLABKOREA, Namyangju, Korea) under a high-purity argon atmosphere. The powder samples were sintered by the hot-press sintering method (Hot-press, Y&I Tech, Paju, Korea) at 773 K for 1 h with a graphite mold (12.7 mm diameter, EANG CARBON, Incheon, Korea). The sintered samples’ densities were about 7.60 g/cm^3^ (~98%), which was close to the calculated density (~7.75 g/cm^3^).

The powder X-ray diffraction (XRD) was performed using Cu-*kα* radiation (D8 advance, Bruker, Karlsruhe, Germany). The polished samples’ microstructure images and elemental mapping were investigated using a high-resolution field-emission scanning electron microscope with an energy-dispersive X-ray (EDX) spectroscopy (MERLIN, Carl Zeiss, Oberkochen, Germany). The electrical conductivity (*σ*) and Seebeck coefficient (*S*) were simultaneously measured by a thermoelectric properties measurement system (ZEM-3, ULVAC-RIKO, Yokohama, Japan) under a helium atmosphere. The Hall mobility *μ_H_* was calculated by the *σ = enμ*, where *e* is the electronic charge and *n* is the Hall carrier density. The Hall carrier density *n_H_* was obtained from the Hall resistivity $\rho_{xy}$ measurement from the relation of *n_H_ = −1/(eR_H_*), *R_H_ = ρ_xy_/H*, where *R_H_* is the Hall coefficient and *H* is the applied magnetic fields ranging from −5 to 5 T. The Hall resistivity was measured by the four-probe contact method using a physical property measurement system (PPMS Dynacool 14T, Quantum Design, San Diego, CA, USA). The thermal conductivity *κ* was calculated from the relation of *κ = λρ_s_C_p_*, where *λ* is the thermal diffusivity measured by a laser flash method (LFA-456, NETZSCH, Selb, Germany), *ρ_s_* is the sample density, and the *C_p_* is the specific heat, which is estimated by the high-temperature extrapolation, measured by the PPMS Dynacool 14T.

## 3. Results

**Figure 1:**
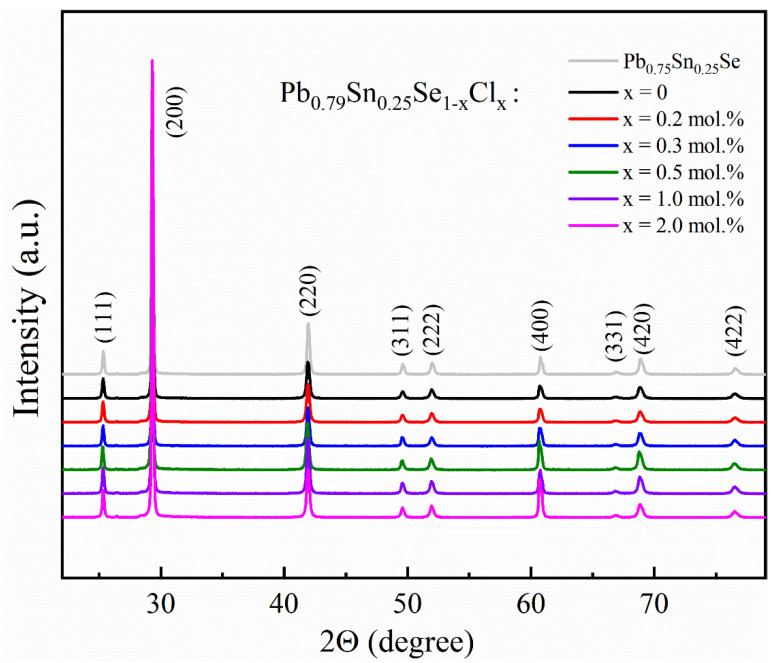
Powder X-ray diffraction (XRD) peaks of the Pb_0.79_Sn_0.25_Se_1−x_Cl_x_ (x = 0.0, 0.2, 0.3, 0.5, 1.0, 2.0 mol.%) and Pb_0.75_Sn_0.25_Se compounds.

The structural characterization of the Pb_0.79_Sn_0.25_Se_1−x_Cl_x_ (x = 0.0, 0.2, 0.3, 0.5, 1.0, 2.0 mol.%) and Pb_0.75_Sn_0.25_Se compounds was performed by the powder XRD, as shown in Figure 1. The XRD peaks of all the samples were indexed by the rock salt-type cubic structure (*Fm-3m*, space group No. 225) without any impurity peaks. The XRD peaks of the Pb-excess and Cl-doped samples were not shifted significantly comparing with the Pb_0.75_Sn_0.25_Se sample. The lattice parameter (a = 6.095 Å) of the samples was comparable with the values (a = 6.085 Å [16], 6.093 Å [17], 6.098 Å [7]) of the Pb_1−x_Sn_x_Se compounds.

**Figure 2:**
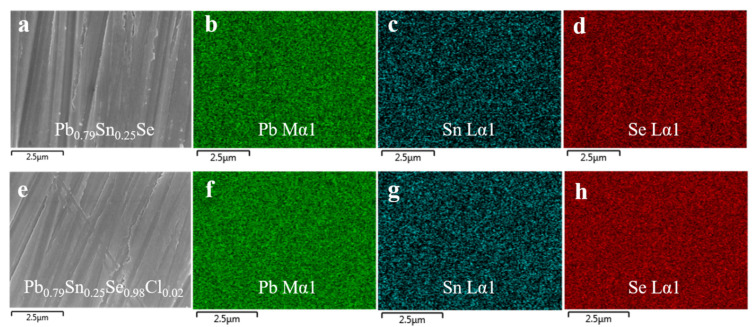
(**a**) Scanning electron microscope (SEM) image of the sintered Pb_0.79_Sn_0.25_Se and elemental maps of (**b**) Pb, (**c**) Sn, and (**d**) Se. (**e**) SEM image of the sintered Pb_0.79_Sn_0.25_Se_0.98_Cl_0.02_ and elemental maps of (**f**) Pb, (**g**) Sn, and (**h**) Se.

The scanning electron microscope (SEM) images of the Pb_0.75_Sn_0.25_Se and Pb_0.79_Sn_0.25_Se_1−x_Cl_x_ (x = 2.0 mol.%) compounds are shown in the Figure 2a,e. The elemental mapping images of the Pb, Sn, and Se for the compounds, measured by the EDX, are shown in Figure 2b–d,f–h, respectively. The SEM/EDX results showed that the sintered samples were homogeneous without any secondary phase or segregation.

**Figure 3:**
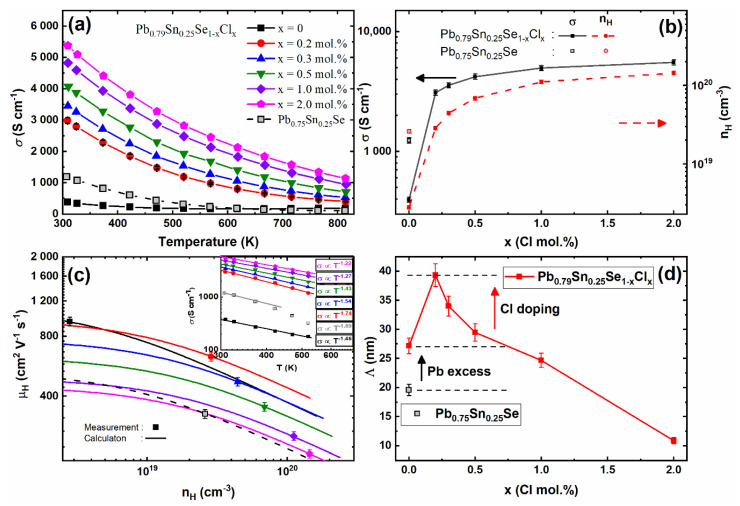
(**a**) Temperature-dependent electrical conductivity *σ(T)*; (**b**) the electrical conductivity (left axis) and Hall carrier concentration (right axis) as a function of the Cl-doping concentration; (**c**) the Hall mobility with the Hall carrier concentration (inset shows *σ(T)* with the linear fitted line by the relation of the *σ* ∝ *T^-n^*); (**d**) the mean free path Λ of the carrier as a function of the Cl-doping concentration of the Pb_0.79_Sn_0.25_Se_1−x_Cl_x_ (x = 0, 0.2, 0.3, 0.5, 1.0, 2.0 mol.%) and Pb_0.75_Sn_0.25_Se compounds at 300 K.

The temperature-dependent electrical conductivities of the Pb_0.79_Sn_0.25_Se_1−x_Cl_x_ (x = 0.0, 0.2, 0.3, 0.5, 1.0, 2.0 mol.%) and Pb_0.75_Sn_0.25_Se compounds are shown in Figure 3a. The electrical conductivity of all sintered samples showed metallic or highly degenerated semiconductor behavior. The Pb-excess sample’s electrical conductivities (Pb_0.79_Sn_0.25_Se) were decreased compared with the non-Pb-excess sample (Pb_0.75_Sn_0.25_Se). The reduced electrical conductivities by the Pb-excess sample were mainly affected by the decreased Hall carrier concentration, as shown in Figure 3b. Since the PbSe has intrinsic vacancy defects [15], the Pb-addition decreases the Hall carrier concentration by the reduction of acceptors and changes the Hall coefficients from positive to negative values, similar to the Pb-addition on PbSe [14]. The electrical conductivities of the Pb-excess and Cl-doped samples (x = 0.0, 0.2, 0.3, 0.5, 1.0, 2.0 mol.%) were increased with the increasing Cl-doping concentration by the enhanced Hall carrier concentration.

The Hall carrier concentration and Hall mobility are affected by the Cl-doping on the Pb_0.79_Sn_0.25_Se as the following relation *σ = neμ* [18]. The theoretical Hall carrier concentration ($n_{H}=n/r_{H})$ and Hall mobility ($\mu_{H}=\mu /r_{H}$) are calculated using the following equations:

(1) $$\mu =\mu_{0}\frac{\left(r+\frac{3}{2}\right)F_{r+\frac{1}{2}}\left(\eta \right)}{F_{\frac{1}{2}}\left(\eta \right)}\;$$

(2) $$n=4\pi {\left(\frac{2m^{*}k_{B}T}{h^{2}}\right)}^{3/2}F_{\frac{1}{2}}\left(\eta \right)$$

(3) $$r_{H}=\frac{3}{2}\frac{\left(2r+\frac{3}{2}\right)}{{\left(r+\frac{3}{2}\right)}^{2}}\frac{F_{\frac{1}{2}}\left(\eta \right)F_{\frac{1}{2}+2r}\left(\eta \right)}{F_{r+\frac{1}{2}}{\left(\eta \right)}^{2}}$$

where *m^∗^*, *r_H_*, *η*, and *F_n_(η)* are the effective mass of the carrier, the Hall factor, the reduced Fermi energy (*η = E_F_/k_B_T*), and the *n-*th order Fermi integral given by $F_{n}\left(\eta \right)=\int_{0}^{\infty }\frac{x^{n}}{1+e^{x-\eta }}dx$, respectively [18].

The carrier’s effective masses are calculated using the measured Hall carrier concentration and the calculated reduced Fermi energy from the experimentally measured Seebeck coefficient with the following equation:

(4) $$S=\pm \frac{k_{B}}{e}\left\{\frac{\left(r+\frac{5}{2}\right)F_{r+\frac{3}{2}}\left(\eta \right)}{\left(r+\frac{3}{2}\right)F_{r+\frac{1}{2}}\left(\eta \right)}-\eta \right\}$$

where *r* is the scattering factor. The main scattering mechanism of the carriers can be estimated roughly from the *σ(T)* with the relation of the *σ* ∝ *T^n^* [19,20]. Because the *n* values of the Pb_0.79_Sn_0.25_Se_1−x_Cl_x_ (x = 0.0, 0.2, 0.3, 0.5, 1.0, 2.0 mol.%) and Pb_0.75_Sn_0.25_Se compounds were close to -1.5 near room temperature, as shown in the inset of Figure 3c, the main scattering mechanism of the samples could be regarded as the acoustic phonon scattering. For acoustic phonon scattering, *r* is −1/2 [18].

The Hall mobilities versus the Hall carrier concentrations of the Pb_0.79_Sn_0.25_Se_1−x_Cl_x_ (x = 0.0, 0.2, 0.3, 0.5, 1.0, 2.0 mol.%) and Pb_0.75_Sn_0.25_Se compounds at 300 K are presented in Figure 3c with the calculated Hall mobilities (lines). The reduction of intrinsic defects increased the calculated Hall mobilities of the Pb-excess samples as compared with the pristine compound (Pb_0.75_Sn_0.25_Se). The calculated Hall mobilities of the Pb-excess and Cl-doped samples (x = 0.0, 0.2, 0.3, 0.5, 1.0, 2.0 mol.%) decreased with the increasing Cl-coping concentration. Even though the Cl-doping can decrease the Hall mobility, the Hall mobilities were not significantly decreased at low Cl-doping concentrations (x = 0.2 and 0.3 mol.%).

The Hall mobility can be affected by the mean free path *Λ* of the carrier, which is calculated by the following equation [21,22]:

(5) $$\Lambda =\sqrt{2E_{F}m^{*}}\frac{\mu }{e}\;$$

(6) $$E_{F}=\left(r+\frac{3}{2}\right)\frac{\pi^{2}k_{B}^{2}T}{3eS}$$

where *E_F_* is the Fermi energy, *m^∗^* is the single-band effective mass of carrier, and *k_B_* is the Boltzmann constant.

The carrier’s mean free path *Λ* is enhanced by the Pb-excess and Cl-doping (at low doping concentrations of x = 0.2, 0.3, and 0.5 mol.%), as shown in Figure 3d. At low Cl-doping concentrations in the Pb-excess samples, the enhanced Hall mobilities and mean free paths of carriers indicated that the Pb-excess and Cl-doping can improve the carrier transport properties on the (Pb,Sn)Se.

**Figure 4:**
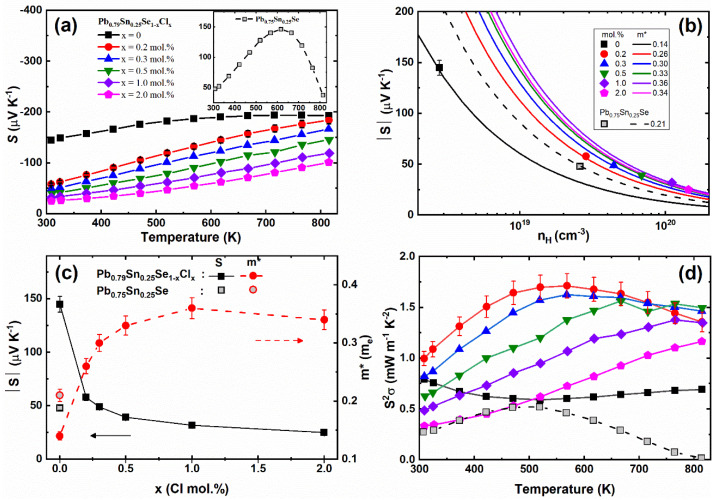
(**a**) Temperature-dependent Seebeck coefficient *S(T)*; (**b**) room temperature Seebeck coefficient as a function of the Hall carrier concentration; (**c**) Seebeck coefficient and effective mass of carrier as a function of the Cl-doping concentration; (**d**) Temperature-dependent power factor *S^2^σ* of the Pb_0.79_Sn_0.25_Se_1−x_Cl_x_ (x = 0.0, 0.2, 0.3, 0.5, 1.0, 2.0 mol.%) and Pb_0.75_Sn_0.25_Se compounds.

Figure 4a and the inset show the temperature-dependent Seebeck coefficients *S(T)* of the Pb_0.79_Sn_0.25_Se_1−x_Cl_x_ (x = 0.0, 0.2, 0.3, 0.5, 1.0, 2.0 mol.%) and Pb_0.75_Sn_0.25_Se compounds, respectively. The Seebeck coefficients of the pristine sample showed the positive values comparable with the positive Seebeck coefficients of Pb_0.7_Sn_0.3_Se [13] and PbSe [14]. The positive Seebeck coefficient of the pristine sample (Pb_0.75_Sn_0.25_Se) was changed to a negative value by the Pb-excess doping (Pb_0.79_Sn_0.25_Se). The sign change of the Seebeck coefficients of the Pb-excess samples can be understood by the carrier’s defect. The density-functional theory shows that the Pb-vacancy of PbSe can act as an acceptor, while the Se-vacancy is a donor [15]. The experimental result is consistent with the theoretical calculation, in that the *p-*type PbSe was changed to the *n-*type by the Pb-addition [14]. The negative Seebeck coefficient of the Pb-excess sample (Pb_0.79_Sn_0.25_Se) corresponds to the previous results. The Seebeck coefficients of the Cl-doped samples (x = 0.2, 0.3, 0.5, 1.0, 2.0 mol.%) decreased with the increasing Cl-doping concentration by the enhanced carrier concentration.

Using Equations (2) and (4), the effective masses of the carrier were obtained and are presented in Figure 4b,c. The decreased effective mass of the carrier of the Pb-excess sample was attributed to the reduced Pb-vacancy defects as compared with the non-Pb-excess sample. The increased effective masses of the carrier by the Cl-doping can be understood by the crystalline mirror symmetry breaking in the Cl-doped Pb_0.7_Sn_0.3_Se [13]. The enhanced carrier scattering by Cl-doping can increase the effective mass of the carrier. As a result, the power factor is significantly enhanced at low Cl-doping concentrations, as shown in Figure 4d. The Pb-excess samples can increase the power factor by the enhanced Seebeck coefficient associated with the reduced Pb-vacancy. However, since the electrical conductivity was also decreased by the reduced carrier concentration, the Pb-excess was not found to be efficient to increase the power factor. Instead, the Cl-doping in the Pb-excess samples (Pb_0.79_Sn_0.25_Se) effectively increased the carrier concentration and increased the effective mass of the carrier by the crystalline mirror symmetry breaking.

**Figure 5:**
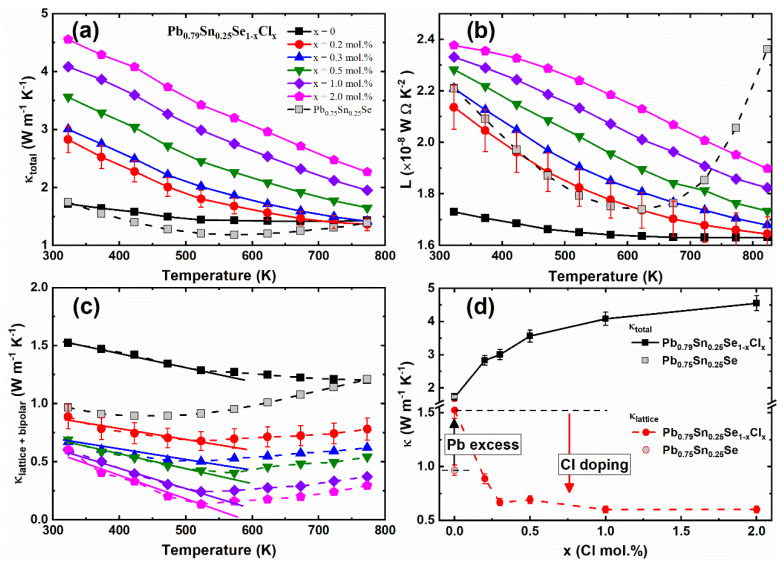
(**a**) Temperature-dependent total thermal conductivity *κ_total_(T);* (**b**) Lorenz number *L(T)*; (**c**) lattice and bipolar thermal conductivity *κ_lattice + bipolar_(T)*; (**d**) total thermal conductivity (black square) and lattice and bipolar thermal conductivity (red circle) as a function of the Cl-doping concentration of the Pb_0.79_Sn_0.25_Se_1−x_Cl_x_ (x = 0.0, 0.2, 0.3, 0.5, 1.0, 2.0 mol.%) and Pb_0.75_Sn_0.25_Se compounds.

The temperature-dependent total thermal conductivities *κ_total_ (T)* of the Pb_0.79_Sn_0.25_Se_1−x_Cl_x_ (x = 0.0, 0.2, 0.3, 0.5, 1.0, 2.0 mol.%) and Pb_0.75_Sn_0.25_Se compounds are presented in Figure 5a. Overall, the *κ_total_* of the non-Pb-excess sample (Pb_0.75_Sn_0.25_Se) was lower than the values of the Pb-excess sample (Pb_0.79_Sn_0.25_Se). The *κ_total_* of the Cl-doped Pb-excess samples (x = 0.2, 0.3, 0.5, 1.0, 2.0 mol.%) increased with the increasing Cl-doping concentration.

To obtain the lattice thermal conductivity, we calculated the Lorenz numbers of the Pb_0.79_Sn_0.25_Se_1−x_Cl_x_ (x = 0.0, 0.2, 0.3, 0.5, 1.0, 2.0 mol.%) and Pb_0.75_Sn_0.25_Se compounds by using Equation (4) and the following equation [23]:

(7) $$L={\left(\frac{k_{B}}{e}\right)}^{2}\left(\frac{\left(r+\frac{7}{2}\right)F_{r+\frac{5}{2}}\left(\eta \right)}{\left(r+\frac{3}{2}\right)F_{r+\frac{1}{2}}\left(\eta \right)}-{\left[\frac{\left(r+\frac{5}{2}\right)F_{r+\frac{3}{2}}\left(\eta \right)}{\left(r+\frac{3}{2}\right)F_{r+\frac{1}{2}}\left(\eta \right)}\right]}^{2}\right)$$

The temperature-dependent Lorenz numbers *L(T)* of the Pb_0.79_Sn_0.25_Se_1−x_Cl_x_ (x = 0.0, 0.2, 0.3, 0.5, 1.0, 2.0 mol.%) and Pb_0.75_Sn_0.25_Se compounds are shown in Figure 5b. The electrical contribution in the total thermal conductivity was obtained by the Wiedemann–Franz law (*κ_el_ = L_0_σT*), where *κ_el_*, *L_0_*, *σ*, and *T* are the electrical thermal conductivity, Lorenz number, electrical conductivity, and absolute temperature, respectively [23].

Figure 5c presents the thermal conductivities of the lattice and bipolar contribution (*κ_lattice + bipolar_*), which were obtained by subtracting the electrical thermal conductivity from the total thermal conductivity. The lattice and bipolar thermal conductivities *κ_lattice + bipolar_* of the Pb_0.75_Sn_0.25_Se slightly decreased with the increasing temperature near room temperature, and then increased by the bipolar effect at high temperature, similar to the bulk PbSe [14]. The lattice and bipolar thermal conductivities *κ_lattice +_* *κ_bipolar_* of the Pb_0.79_Sn_0.25_Se_1−x_Cl_x_ (x = 0.0, 0.2, 0.3, 0.5, 1.0, 2.0 mol.%) also showed the conventional *κ ~ 1/T* behavior below 525 K and the bipolar effect at high temperature (*T* ≥ 550 K). The Umklapp processes of acoustic phonon mainly cause the *1/T* behavior of the thermal conductivity at high temperature [24]. The bipolar effect is associated with the small bandgap of the PbSe [14]. A previous study showed that the Cl-doping suppresses the bipolar effect in Pb_0.7_Sn_0.3_Se_1−x_Cl_x_ because of the bandgap [13]. On the other hand, the bipolar effect was not suppressed by the Cl-doping in this result. This indicates that the bipolar effect depends on the Pb-excess rather than the Cl-doping. It suggests that the bipolar effect of the Pb_0.79_Sn_0.25_Se_1−x_Cl_x_ (x = 0.0, 0.2, 0.3, 0.5, 1.0, 2.0 mol.%) and Pb_0.75_Sn_0.25_Se compounds are related with the minority carrier excitation, rather than the bandgap energy.

As compared with the non-Pb-excess sample (Pb_0.75_Sn_0.25_Se), the *κ_lattice +_* *κ_bipolar_* of the Pb-excess sample (Pb_0.79_Sn_0.25_Se) were enhanced, and the *κ_lattice +_* *κ_bipolar_* of the Cl-doped Pb-excess samples decreased with the increasing Cl-doping concentration, as shown in Figure 5d. In contrast, the Pb-excess increased the *κ_lattice +_* *κ_bipolar_* by the Pb-vacancy defect reduction, and the *κ_lattice +_* *κ_bipolar_* of the Cl-doped samples significantly decreased by the crystalline mirror symmetry breaking.

**Figure 6:**
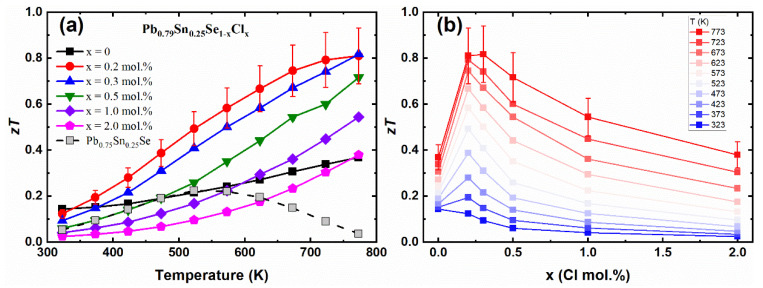
(**a**) Temperature-dependent *zT* values; (**b**) The *zT* values for the various temperatures, as indicated, with the Cl-doping concentration of the Pb_0.79_Sn_0.25_Se_1−x_Cl_x_ (x = 0.0, 0.2, 0.3, 0.5, 1.0, 2.0 mol.%) and Pb_0.75_Sn_0.25_Se compounds.

Figure 6a shows the temperature-dependent dimensionless figure of merit *zT* values of the Pb_0.79_Sn_0.25_Se_1−x_Cl_x_ (x = 0.0, 0.2, 0.3, 0.5, 1.0, 2.0 mol.%) and Pb_0.75_Sn_0.25_Se compounds. The *zT* values of the Pb-excess sample (Pb_0.79_Sn_0.25_Se) were higher than the values of the non-Pb-excess sample (Pb_0.75_Sn_0.25_Se) by the enhanced Hall mobility. The *zT* values of the Cl-doped Pb-excess samples were significantly enhanced as compared with the non-Cl-doped samples. The maximum *zT* values of the Pb_0.79_Sn_0.25_Se_1−x_Cl_x_ (x = 0.2, 0.3 mol.%) were 0.81 and 0.82 (at 773 K), respectively, which was a significantly increased value compared to the *zT* = 0.64 (at 823 K) of the similar composition (Pb_0.7_Sn_0.3_Se_0.99_Cl_0.01_) [13]. The *zT* values of the Pb_0.79_Sn_0.25_Se_1−x_Cl_x_ (x = 0.2, 0.3 mol.%) clearly show that the Pb-excess and Cl-doping are effective to increase the thermoelectric performance of the PbSe-based compounds.

## 4. Conclusions

In summary, the thermoelectric properties of the sintered bulk samples of the Pb_0.79_Sn_0.25_Se_1−x_Cl_x_ (x = 0.0, 0.2, 0.3, 0.5, 1.0, 2.0 mol.%) and Pb_0.75_Sn_0.25_Se compounds were investigated. The electrical resistivity and the Hall carrier concentration results clearly show that the Cl-doping in the Pb-excess (Pb,Sn)Se samples can increase the carrier concentration. Additionally, the relatively high Hall mobility is possible by the carrier’s enhanced mean free path at low Cl-doping concentrations (below 0.3 mol.%). The Seebeck coefficients decreased with the increasing Cl-doping concentration and carrier concentration. On the other hand, the carrier’s enhanced effective masses can be understood by the crystalline mirror symmetry breaking from Cl-doping. From the thermal conductivity measurements, the crystalline mirror symmetry breaking by the Cl-doping can significantly decrease the lattice thermal conductivity of the Pb-excess (Pb,Sn)Se. Furthermore, the bipolar effect was suppressed in the Pb-excess samples. As a result, the *zT* values of x = 0.2 and 0.3 mol.% increased up to 0.8 at 773 K. Therefore, we suggest that Pb-excess and crystal mirror symmetry breaking by Cl-doping effectively increases the thermoelectric performance in the (Pb,Sn)Se system.

## Data Availability

The data presented in this study are available on request from the corresponding author.
